# Maternal vitamin D supplementation during pregnancy and lactation to prevent acute respiratory infections in infancy in Dhaka, Bangladesh (MDARI trial): protocol for a prospective cohort study nested within a randomized controlled trial

**DOI:** 10.1186/s12884-016-1103-9

**Published:** 2016-10-13

**Authors:** Shaun K. Morris, Lisa G. Pell, Mohammed Ziaur Rahman, Michelle C. Dimitris, Abdullah Mahmud, M. Munirul Islam, Tahmeed Ahmed, Eleanor Pullenayegum, Tahmid Kashem, Shaila S. Shanta, Jonathan Gubbay, Eszter Papp, Michelle Science, Stanley Zlotkin, Daniel E. Roth

**Affiliations:** 1 Department of Paediatrics, University of Toronto and the Hospital for Sick Children, 555 University Avenue, Toronto, ON Canada; 2 Centre for Global Child Health, SickKids Research Institute, Hospital for Sick Children, 686 Bay Street, Toronto, ON Canada; 3 Child Health Evaluative Sciences, SickKids Research Institute, Hospital for Sick Children, 686 Bay Street, Toronto, ON Canada; 4 Zoonotic Diseases Research Group, Centre for Communicable Diseases, icddr,b, 68 Shaheed Tajuddin Ahmed Sarani, Mohakhali, Dhaka, 1212 Bangladesh; 5 Centre for Child and Adolescent Health, icddr,b, 68 Shaheed Tajuddin Ahmed Sarani, Mohakhali, Dhaka, 1212 Bangladesh; 6 Centre for Nutrition and Food Security, icddr,b, 68 Shaheed Tajuddin Ahmed Sarani, Mohakhali, Dhaka, 1212 Bangladesh; 7 Public Health Ontario, 661 University Ave., Toronto, ON Canada; 8 Division of Infectious Diseases, Hospital for Sick Children, 555 University Avenue, Toronto, ON M5G1X8 Canada

**Keywords:** Vitamin D, Pregnancy, Infant, Acute respiratory infection, Pneumonia, Influenza, Respiratory syncytial virus, *Streptococcus pneumoniae*, Bangladesh

## Abstract

**Background:**

Early infancy is a high-risk period for severe acute respiratory infection (ARI), particularly in low-income countries with resource-limited health systems. Lower respiratory tract infection (LRTI) is commonly preceded by upper respiratory infection (URTI), and often caused by respiratory syncytial virus (RSV), influenza and other common community-acquired viral pathogens. Vitamin D status is a candidate modifiable early-life determinant of the host antiviral immune response and thus may influence the risk of ARI-associated morbidity in high-risk populations.

**Methods/Design:**

In the Maternal Vitamin D for Infant Growth (MDIG) study in Dhaka, Bangladesh (NCT01924013), 1300 pregnant women are randomized to one of five groups: placebo, 4200 IU/week, 16,800 IU/week, or 28,000 IU/week from 2^nd^ trimester to delivery plus placebo from 0–6 months postpartum; or, 28,000 IU/week prenatal and until 6-months postpartum. In the Maternal Vitamin D for ARI in Infancy (MDARI) sub-study nested within the MDIG trial, trained personnel conduct weekly postnatal home visits to inquire about ARI symptoms and conduct a standardized clinical assessment. Supplementary home visits between surveillance visits are conducted when caregivers make phone notifications of new infant symptoms. Mid-turbinate nasal swab samples are obtained from infants who meet standardized clinical ARI criteria. Specimens are tested by polymerase chain reaction (PCR) for 8 viruses (influenza A/B, parainfluenza 1/2/3, RSV, adenovirus, and human metapneumovirus), and nasal carriage density of *Streptococcus pneumoniae.* The primary outcome is the incidence rate of microbiologically-positive viral ARI, using incidence rate ratios to estimate between-group differences. We hypothesize that among infants 0–6 months of age, the incidence of microbiologically-confirmed viral ARI will be significantly lower in infants whose mothers received high-dose prenatal/postpartum vitamin D supplements versus placebo. Secondary outcomes include incidence of ARI associated with specific pathogens (influenza A or B, RSV), clinical ARI, and density of pneumococcal carriage.

**Discussion:**

If shown to reduce the risk of viral ARI in infancy, integration of maternal prenatal/postpartum vitamin D supplementation into antenatal care programs in South Asia may be a feasible primary preventive strategy to reduce the burden of ARI-associated morbidity and mortality in young infants.

**Trial registration:**

NCT02388516, registered March 9, 2015.

## Background

Early infancy is a high-risk period for severe acute respiratory infection (ARI), particularly in low-income countries with resource-limited health systems. Lower respiratory tract infection (LRTI) accounts for more deaths among children under the age of 5 in the world than any other single disease [1]. Notably, the clinical course of a LRTI often starts with an upper respiratory tract infection (URTI) suggesting an important role of URTI in the pathogenesis of more severe and life-threatening LRTI. Respiratory syncytial virus (RSV) and influenza are common viral causes of both URTI and LRTI in young children. The treatment of RSV and influenza along with other viral infections remains unsatisfactory and primary prevention remains a key strategy in reducing the burden of these infections.

Vitamin D has been linked to the risk of respiratory infections such as pneumonia, tuberculosis and bronchiolitis, suggesting it may be a potentially modifiable determinant of the host immune response. It has been postulated that lower vitamin D levels may explain the seasonal variation in influenza and studies have shown that children with rickets are at increased risk of lower respiratory tract disease or pneumonia [2–4]. Higher rates of invasive pneumococcal disease have been associated with decreased ultraviolet light exposure through the postulated mechanism of lower vitamin D levels [5]. Vitamin D has been shown to modulate the innate immune response in lung epithelial cells [6, 7], and antimicrobial peptides induced by vitamin D may have antiviral effects based on observed activity against herpes simplex virus-1 (HSV-1) [8], adenovirus [9], human immunodeficiency virus (HIV) [10] and vaccinia [11]. In RSV-infected human airway epithelial cells, vitamin D induces Iκβα, an NF-κB inhibitor, in airway epithelium and decreases RSV induction of inflammatory genes [7].

Several observational studies have evaluated the association of serum 25-hydroxyvitamin D (25 (OH) D) concentration with incidence or severity of respiratory tract infections. Studies in adults have shown an association between lower 25 (OH) D concentrations and increased URTI (self-reported) [12–14] and absence from work due to respiratory symptoms [12, 15]. Pediatric studies have focused predominantly on LRTI (chest X-ray confirmed pneumonia or bronchiolitis) [16–20]. These studies have shown an association between low serum 25 (OH) D levels and risk of LRTI in children in India [16], Bangladesh [19] and Turkey [17]. More recently, a study in Hutterite communities in Canada found an association between serum 25 (OH) D level and laboratory confirmed URTI [21].

Despite these suggestive results from observational studies, there have been few prospective randomized controlled trials examining the impact of vitamin D supplementation on risk of ARI in children. In a trial in Japan, school children randomized to 1200 IU/day of vitamin D compared to placebo had a significantly decreased risk of developing laboratory-proven influenza A (RR 0.58, 95 % CI 0.34, 0.99) but not influenza B (RR 1.39, 95 % CI 0.90-2.15) [22]. Similarly, a decrease in parent-reported ARI was noted in children in Mongolia given vitamin D-fortified milk (300 IU of vitamin D_3_) [23], in a setting in which children had very low baseline serum 25 (OH) D levels. However, a trial in Afghanistan involving over 3000 children randomized to receive quarterly doses of 100,000 IU of vitamin D or placebo found no difference in the incidence of pneumonia [24].

The first randomized trial of prenatal vitamin D supplementation on childhood wheeze was recently published [25]. In this study, pregnant women in London, England were randomized at 27 weeks gestation to receive either no vitamin D, 800 IU ergocalciferol daily until delivery, or a single bolus of 200,000 IU cholecalciferol. This study found no difference in ‘wheeze ever’ between groups nor any difference in prevalence in LRTIs after adjusting for multiple testing. However, the researchers identified multiple limitations in their study including lack of blinding of participants and subjective outcomes, small sample size and limited statistical power, and dosages of vitamin D that lead to only 13 % of the daily group and 3 % of the bolus group having cord serum 25 (OH) D levels considered to be in a sufficient range.

The Maternal Vitamin D for ARI (MDARI) study addresses important gaps in the vitamin D and respiratory tract infection literature. First, there have been no studies examining the effect of prenatal vitamin D supplementation on a primary outcome of laboratory-confirmed viral respiratory infections in the first 6 months of life, a high-risk period for both vitamin D deficiency and respiratory tract infections. Second, the role of prenatal vitamin D as a determinant of susceptibility to specific viral infections has not been examined. Third, there have been no studies assessing the association between vitamin D supplementation and quantitative nasal carriage of *Streptococcus pneumoniae* (pneumococcus), the most important cause of bacterial LRTI in young children worldwide.

In South Asia, biochemical vitamin D deficiency is common among women and young infants [26]. Our study in Dhaka is being conducted in a population known to have a high prevalence of vitamin D deficiency as well as high incidence of early infant respiratory infection morbidity and mortality [27–29].

This study is integrated into a randomized clinical trial of maternal vitamin D supplementation for which the primary outcome is length at one year of age (Maternal Vitamin D for Infant Growth (MDIG) Trial, Clinicaltrials.org identifier NCT01924013), currently underway in Dhaka, Bangladesh and funded by the Bill and Melinda Gates Foundation (Achieving Health Growth Platform Grant OPP1066764) [30]. We hypothesize that among infants 0–6 months, the incidence of microbiologically confirmed ARI will be significantly lower in infants of mothers who received a relatively high dose of vitamin D (28,000 IU/week) during pregnancy plus continued postpartum supplementation (to 6 months postpartum) compared to mothers who received placebo throughout the prenatal and postpartum periods. If a significantly decreased incidence of microbiologically confirmed ARI is found, we will further assess for effect of lower doses of prenatal maternal vitamin D supplementation. We aim to quantify the effect of maternal vitamin D supplementation on the relative incidence of, a) microbiologically confirmed ARI (primary outcome); b) ARI due to influenza (A&B) and RSV; c) clinical ARI; and, d) *S. pneumococcus nasal* carriage density.

## Methods

### Design

The MDIG trial is a randomized placebo-controlled dose-ranging trial of maternal vitamin D supplementation during pregnancy and lactation to improve infant linear growth in Dhaka, Bangladesh. The detailed methods of the MDIG trial are published elsewhere [30]. The research group is primarily a collaboration between icddr,b (International Centre for Diarrhoeal Disease Research, Bangladesh) and the Hospital for Sick Children (Toronto, Canada). Pregnant women were enrolled beginning on March 18, 2014 and enrolment is expected to proceed until August 2015. Enrolment of infants in the MDARI sub-study began on December 2, 2014 and will continue until all infants born in the MDIG trial have been offered enrolment (expected completion by January 2016).

### Setting

The single-site trial is being conducted in Dhaka. Microbiologic testing is being conducted at the Virology Laboratory of icddr,b. Enrolment and clinical activities of the MDIG trial is based at the Maternal and Child Health Training Institute (MCHTI), commonly known as Azimpur Maternity Center, a government facility that provides low-cost health care to pregnant women and children in its referral area in central Dhaka. Full details regarding the trial site geography and population are provided in the MDIG trial methods paper [30].

### Participants

All infants born to women enrolled in the MDIG trial are eligible for the MDARI sub-study. Maternal inclusion criteria for the MDIG trial include being at least 18 years of age, 17 to 24 completed weeks of gestation based on a combination of recalled last menstrual period (LMP) and ultrasound, according to a conventional algorithm (per the Society of Obstetricians and Gynecologists of Canada), and intention to permanently reside in the trial catchment area for the duration of the study. Participants are informed of the ARI sub-study by a research study physician at a clinical visit after enrolment in the main trial. We obtain separate written informed consent for participation in the MDARI sub-study. We aim to enroll at the 30 week gestational age study visit, however, given that the MDIG trial began before MDARI, we also enroll at prenatal visits after 30 weeks, at the time of delivery, and during home visits to infants who were younger than 6 months of age at the time MDARI launched. Participants are free to withdraw from MDARI while remaining in MDIG but withdrawal from MDIG results in automatic withdrawal from MDARI.

### Interventions and randomization

**Fig. 1:**
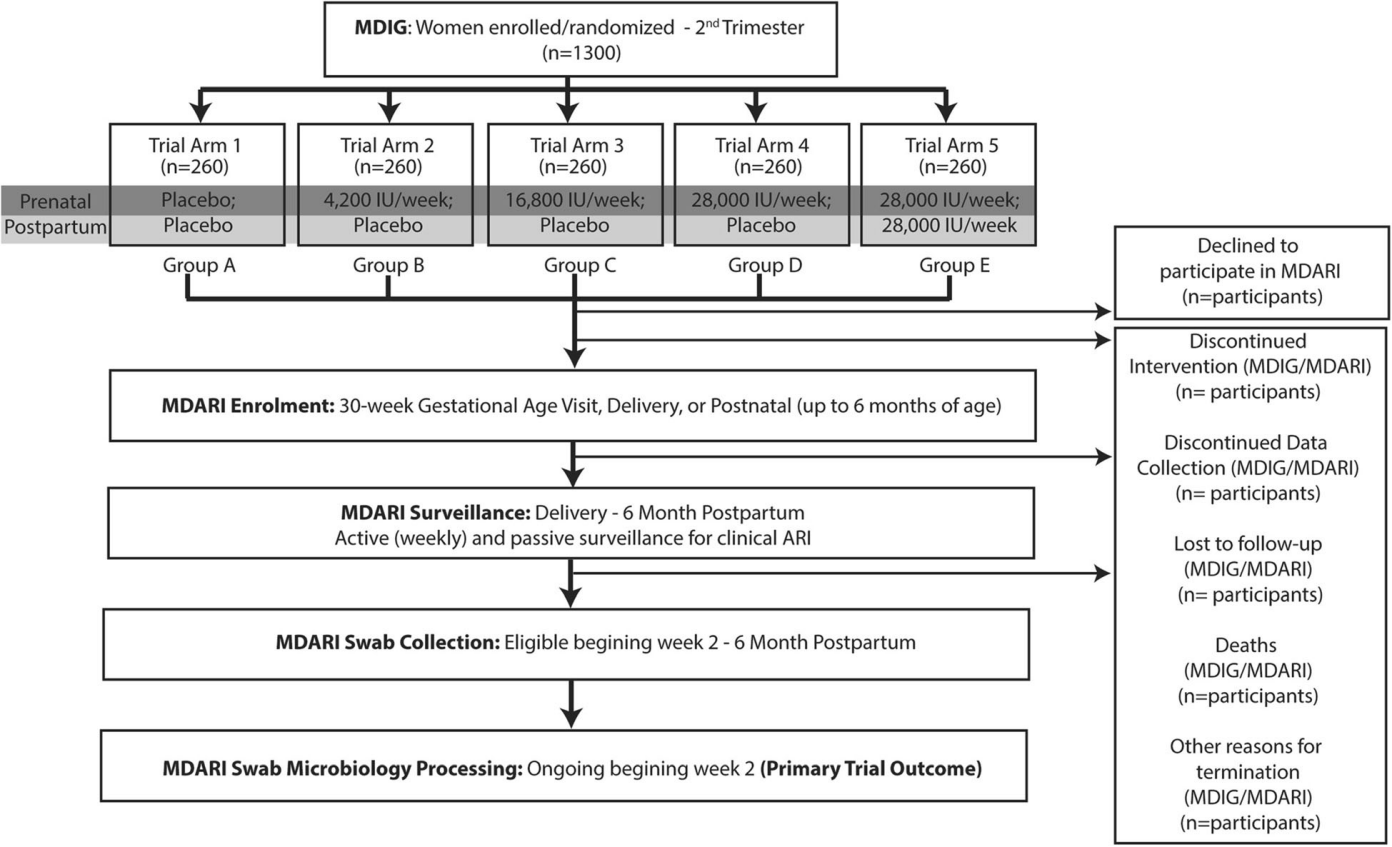
Maternal Vitamin D Acute Respiratory Infection (MDARI) trial flow diagram

A full description of the intervention groups along with the rationale for choice of vitamin D dosages may be found in the methods paper describing the MDIG trial [30]. Three intervention groups are receiving weekly prenatal vitamin D supplementation of 4200 IU/week, 16,800 IU/week, or 28,000 IU/week respectively given as a single weekly dose beginning in the 2^nd^ trimester of pregnancy. These three groups all receive placebo post-partum. A fourth group is receiving 28,000 IU/week during pregnancy and will continue with this level of supplementation until 26 weeks postpartum. A fifth control group is receiving placebo both pre and post-partum (Fig. 1). Infant dietary patterns are assessed at weekly visits to assess breastfeeding patterns and timing of introduction and frequency of consumption of specific complementary foods.

Most infants born to mothers in the placebo and 4200 IU/week groups are not expected to demonstrate neonatal 25 (OH) D ≥ 50 nmol/L, whereas most infants in the 16,000 IU/week and the 28,000 IU/week groups (including participants that will continue to receive 28,000 IU/week post-delivery) are expected to have 25 (OH) D ≥ 50 nmol/L in early infancy. Although the clinical significance of the 50 nmol/L threshold in early infancy remains to be established, this is the cut-off corresponding to the recommended dietary allowance established by the Institute of Medicine in 2011 for all age groups [31]. To promote optimal regimen adherence, doses are delivered weekly (e.g., 28,000 IU per week to deliver the approximate equivalent of 4000 IU/day) and supplementation is directly observed by study personnel whenever feasible.

### Design

This is a prospective cohort study nested within a randomized, placebo-controlled trial with concealment of allocation and blinding throughout intervention and analysis. In the main MDIG trial, a total of 1,300 pregnant women will be randomized, targeting 260 women in each group (Fig. 1). The allocation sequence was generated by the trial statistician using a computer-generated random number sequence, according to a simple randomization scheme. Participants, investigators, field personnel, study lab staff and data analysts are blinded to vitamin D or placebo group allocation. Consent to the MDIG trial is necessary to be eligible for the MDARI study; however, participants in MDIG may choose to decline participation in MDARI.

### Study outcomes

The primary study outcome is microbiologically confirmed ARI. Main secondary outcomes include ARI with microbiologic confirmation of influenza A, influenza B, or RSV; clinical ARI; and quantitative nasal *S. pneumoniae* carriage density. The clinical case definitions for ARI, URTI, and LRTI are shown in Table 1. When an infant meets any of the sets of criteria for clinical URTI or LRTI, nasal swab collection is indicated. All nasal swabs are optimally collected within 24 h of notification, however a nasal swab may still be collected within 7 days following clinical case ascertainment (or within 7 days of hospital discharge) [32–34]. No participant has more than 1 nasal swab collected within 7 days, regardless of the severity of the illness. Swabs are tested by polymerase chain reaction (PCR) for a panel of respiratory viruses consisting of influenza A, influenza B, RSV, parainfluenza 1, 2, and 3, adenovirus, and human metapneumovirus. Swabs are also be tested for quantitative *S. pneumoniae* carriage density. Full details of microbiologic testing are presented below. To be considered an incident case, there must be either a preceding surveillance week in which a clinical case definition was not met or a worsening of a case from URTI to LRTI or from LRTI to hospitalized LRTI. To avoid including common neonatal problems that may present similarly to ARI (e.g., transient tachypnea of the newborn), case definitions cannot be met in the first 7 days of life.

**Table 1:** Study outcome definitions

Acute Respiratory Infection (ARI)	Any upper respiratory tract infection or lower respiratory tract infection as defined below
Clinical Upper Respiratory Tract Infection (URTI)	A new-onset illness consisting of at least two of the following clinical criteria at any time during a surveillance week: • Caregiver-reported cough; • Caregiver-reported rhinorrhea; • Caregiver-reported nasal congestion; • Measured temperature ≥37.5 °C (axillary) confirmed with 2nd measurement
Clinical Lower Respiratory Tract Infection (LRTI)	• Caregiver-reported cough AND/OR difficulty breathing during a surveillance week AND • Observed elevated respiratory rate (60 breaths per minute or greater for infant up to 59 days of age, or 50 breaths per minute or greater for infant 60 days of age or older) and/or lower chest wall in-drawing OR • Hospitalization with physician diagnosis of pneumonia or bronchiolitis
Microbiologically Confirmed URTI	URTI that tests positive for at least one of influenza A, influenza B, respiratory syncytial virus, parainfluenza 1, 2, and 3, adenovirus, or human metapneumovirus
Microbiologically Confirmed LRTI	LRTI that tests positive for at least one of influenza A, influenza B, respiratory syncytial virus, parainfluenza 1, 2, and 3, adenovirus, or human metapneumovirus

### Surveillance of acute respiratory infections

Active and passive surveillance mechanisms are being utilized to prospectively identify new cases of clinical ARI among infants during the 0–6 month postnatal period.

Active surveillance visits are attempted on a weekly basis during the first 6 months of life and are implemented through the existing design and infrastructure of the MDIG study [30]; the majority of these visits occur in participants’ homes. At each surveillance-visit, study field personnel trained according to the Integrated Management of Childhood Illness (IMCI) program (WHO/UNICEF) [35] administer a standardized questionnaire and conduct an infant-clinical examination. The questionnaire and physical exam have been designed to optimize the collection of maternal-reported and CHW-observed infant morbidity data. Information pertaining to the following ARI-related symptoms is collected: 1) maternal-reported cough, rhinorrhea (i.e., runny nose), nasal congestion (i.e., stuffy nose), difficulty breathing, and infant-hospitalizations related to a breathing problem or chest infection (e.g., pneumonia); and, 2) CHW-observed axillary temperature ≥ 37.5 °C (confirmed with a second measurement), lower chest wall indrawing, and elevated respiratory rate (≥60 breaths per minutes for infants up to 59 days of age or ≥ 50 breaths per minute for infants 60 days of age or older). The routine collection of these data facilitates the weekly identification of new, worsened, or persistent cases of ARI. The identification of any one symptom indicative of a possible ARI during weekly surveillance visits triggers a field worker to notify the Nasal Swab Dispatcher (MDARI study physician). The Nasal Swab Dispatcher assesses the information to determine whether the clinical case definition of URTI and/or LRTI has been met, and whether a nasal swab specimen should be collected (i.e., no nasal swab collected within past 7 days, at least one study visit at which URTI and LRTI were absent or if not, the ARI clinical case definition has worsened since the last nasal swab was collected).

To identify cases of ARI that arise between scheduled visits, a passive surveillance system has been implemented that relies on the report of ARI symptoms by parents/guardians. At enrolment, mothers are instructed to notify the Nasal Swab Dispatcher by telephone if their infant develops any one of the following symptoms: runny nose, nasal congestion, cough, difficulty breathing, feels hot to the touch, or the infant has been hospitalized. An incentive of approximately $0.25 USD of cell phone credit is provided to families who report an ARI-related symptom. The Nasal Swab Dispatcher assesses the information to evaluate whether the ARI clinical case definition has possibly been met (at least one of cough, runny nose, congestion, hot to the touch, difficulty breathing, or ARI-related hospitalization) and whether collection of a nasal swab is possible (i.e., no nasal swab collected within past 7 days, at least one study visit at which URTI and LRTI were absent or if not, a reassessment must be conducted to determine whether the ARI clinical case definition has worsened since the last nasal swab was collected). Caregiver-reported ARI symptoms are verified in-person through a short questionnaire and infant-clinical examination conducted by a trained nasal swab collector; the clinical assessment is typically performed in the participants’ home. A nasal swab specimen will only be collected if the clinical criteria for URTI and/or LRTI are verified. All MDIG-study physicians have been trained to notify the Nasal Swab Dispatcher if any symptom indicative of a possible ARI is detected during an interaction with an enrolled infant. The Nasal Swab Dispatcher assesses the physician-reported information to determine whether the clinical case definition of an ARI has been met. If an infant is hospitalized with a physician diagnosis of pneumonia or bronchiolitis, the nasal swab collector collects a swab during the hospitalization, or if this is not possible, within 7 days of discharge.

### Pulse oximetry

Measures of infant peripheral oxygen saturation (SpO_2_) are collected for every infant that meets or possibly meets the clinical case definition of an ARI. SpO_2_ is measured with a hand-held pulse oximeter device (Rad-5v with LNCS Y-shaped sensor and LNC-04 connector cable, Masimo California, USA). A threshold of SpO_2_ < 90 % is used to guide the referral of infants to hospital for further assessment.

### Microbiologic specimen collection

Among infants meeting the definition for clinical ARI as described above, the study worker obtains parental consent to perform nasal swabbing on the infant using sterile flocked swabs (Copan Diagnostics, Inc., model number 56780CS01) to obtain nasal epithelial cells for laboratory analysis. Nasal flocked swabs, which are inserted more proximately in the nares than a traditional nasopharyngeal swab, are better tolerated by the child, easier to perform for the health care worker, and reliably provide adequate sample for molecular testing for respiratory viruses [36–38]. Testing is performed by swabbing the nares to the level of the nasal turbinate to obtain a sample of exfoliated cells from the mucosal lining. The infant’s head is tilted backwards to an angle of 70° and the swab is gently inserted by the study worker horizontally into the nasal passage approximately one-half the distance from the nostril to the earlobe or until the stopper meets the infant’s nose or until resistance is met at the level of the turbinate. The swab is then rotated two to three times and held it place for 5–10 s to absorb sample material. The swab is then removed and immediately placed into a collection vial labeled with the patient’s study number and containing 1–3 ml of universal transport medium (Copan Diagnostics, Inc.). The vial is placed into an insulated cold bag and transported to a −80 °C study freezer for storage until testing is done.

Only one nasal swab will be collected for each episode of ARI and there will not be more than 1 swab collected in a 1-week period. We estimate that most infants will not receive more than about one swab per month until they reach 6 months of age, and typically we expect an infant to have 3–4 swabs during this period. Participant families receive 200 taka (~$2.50 USD) for the time involved in the nasal swab procedure.

### Laboratory analysis

Respiratory samples are processed and analyzed in the Virology Lab at icddr,b in Dhaka. For each nasal swab specimen, two aliquots are prepared; one aliquot is processed for real-time assays and one aliquot is stored at −80 °C for future reference. Total nucleic acids are extracted from sample using InviMag Pathogen Kit/KF96 (STRATEC Molecular, Berlin, Germany) to an eluate volume of 200 μl according to the manufacturer’s instructions. The InviMag Pathogen Kit/ KF96 contains the lyophilized lysis component (lysis buffer, Proteinase K and Carrier RNA) for bacterial DNA, viral DNA and viral RNA isolation. Lysed samples are mixed with magnetic beads and nucleic acids are isolated according to the manufacturer’s instructions. All real time PCR tests undergo a visual check; if quality of the amplification is not good the test is repeated and/or considered negative. A house-keeping gene, RNP, is used as an indicator of perfect nucleic acid extraction, to check for epithelial cells, sample quality, quality of RT-PCR, and to monitor for PCR inhibition.

To detect and quantitate pneumococcal copy number, quantitative real-time PCR (qPCR) assays are performed to amplify *lytA*, a single-copy autolysin gene that is specific to all *S. pneumoniae* strains (Table 2). This method is based on the U.S. Centers for Disease Control and Prevention (CDC) method as described by Carvalho et al., and adapted from the National Institute of Communicable Diseases (NICD) in South Africa. The qPCR assays are carried out in a final reaction volume of 25 μl, each containing 10 μl of extracted total nucleic acid. The *lytA* primers and probes are added to a final concentration of 10 μM. DNA is amplified in a ABI 7500 (Applied Biosystem, USA) using the following cycling parameters: 95 °C for 10 min followed by 40 cycles of 95 °C for 15 s and 60 °C for 1 min. All standards are performed in triplicate and a negative (no template) and positive (*lytA* plasmid control) control is included in every run along with the samples. *lytA* amplification data is analyzed using 7500 software version 2.0.6 (Applied Biosystem, USA). The baseline and threshold are manually set at >0.05, the slope of the standard curve is between −3.1 to −3.6 and correlation should be >0.9. The cutoff value CT < 35 is considered as positive. A CT value of ≤38 showing appropriate amplification on visual check is also considered a positive result.

**Table 2:** Primers and probes used in this study for qPCR reactions

Primer Name		Primer/probe Sequence (5′-3′) Nucleotide Sequence 5′-3′
*lytA*	Forward	ACG CAA TCT AGC AGA TGA AGC A
	Reverse	TCG TGC GTT TTA ATT CCA GCT
	Probe (Pb 400)	6FAM-TGC CGA AAA CGC TTG ATA CAG GGA G-MGB
InfA	Forward	GAC CRA TCC TGT CAC CTC TGA C
	Reverse	AGG GCA TTY TGG ACA AAK CGT CTA
	Probe1	6FAM-TGC AGT CCT CGC TCA CTG GGC ACG-BHQ1
InfB	Forward	TCC TCA AYT CAC TCT TCG AGC G
	Reverse	CGG TGC TCT TGA CCA AAT TGG
	Probe1	6FAM-CCA ATT CGA GCA GCT GAA ACT GCG GTG-BHQ1
RNP	Forward	AGA TTT GGA CCT GCG AGC G
	Reverse	GAG CGG CTG TCT CCA CAA GT
	Probe1	6FAM-TTC TGA CCT GAA GGC TCT GCG CG-BHQ1
RSV	Forward	GGC AAA TAT GGA AAC ATA CGT GAA
	Reverse	TCT TTT TCT AGG ACA TTG TAY TGA ACA G
	Probe1	6FAM-CTG TGT ATG TGG AGC CTT CGT GAA GCT-BHQ1
HMPV	Forward	CAA GTG TGA CAT TGC TGA YCT RAA
	Reverse	ACT GCC GCA CAA CAT TTA GRA A
	Probe 1	6FAM-TGG CYG TYA GCT TCA GTC AAT TCA ACA GA-BHQ1
HPIV1	Forward	ACA AGT TGT CAA YGT CTT AAT TCR TAT
	Reverse	TCG GCA CCT AAG TAR TTY TGA GTT
	Probe	6FAM-ATA GGC CAA AGA “T”TG TTG TCG AGA CTA TTC CAA
HPIV2	Forward	GCA TTT CCA ATC TAC AGG ACT ATG A
	Reverse	ACC TCC TGG TAT AGC AGT GAC TGA AC
	Probe	6FAM-CCA TTT ACC “T”AA GTG ATG GAA TCA ATC GCA AA
HPIV3	Forward	TGG YTC AAT CTC AAC AAC AAG ATT TAA G
	Reverse	TAC CCG AGA AAT ATT ATT TTG CC
	Probe1	6FAM-CCC RTC TG“T” TGG ACC AGG GAT ATA CTA CAA A
Adeno	Forward	GCC CCA GTG GTC TTA CAT GCA CAT C
	Reverse	GCC ACG GTG GGG TTT CTA AAC TT
	Probe	6FAM-TG CAC CAG ACC CGG GCT CAG GTA CTC CGA-BHQ1

*Note*: Quenched internally at a modified “T” residue with BHQ1 and a terminal phosphate at the 3′-end to prevent probe extension by Taq polymerase

Individual real-time one step reverse transcriptase-PCR (qRT-PCR) assays are being used to detect influenza A and B viruses. Primer and probe sequences were designed on the basis of CDC recommendations (Table 2). The qRT-PCR assays are carried out in a final reaction volume of 25 μl, each containing 5.0 μl of extracted nucleic acid. The specific influenza A and B primers and probes are added to a final concentration of 20 μM and 5.0 μM respectively. The qRT-PCR reaction is carried out with any of the following machines; ABI 7500 (Applied Biosystem, USA), CFX-96 (Bio-Rad, USA) or LightCycler 480 (Roche, Switzerland) using the following cycling parameters: 50 °C for 30 min, 95 °C for 5 min, 46 cycles at 95 °C for 15 s and 55 °C for 1 min. A negative control (no template) and a CDC provided positive control is included in every run along with the samples. The amplification data is analyzed using machine specific softwares. The cutoff value CT < 35 is considered positive. A CT value of ≤38 showing appropriate amplification on visual check is also be considered a positive result.

Individual qRT-PCR assays for 6 viruses (adenovirus, human metapneumovirus [HMPV], human parainfluenza viruses 1–3 and respiratory syncytial virus [RSV]) are being carried out according to the procedure described by Weinberg et al. [39]. Primer and probe sequences were designed on the basis of CDC recommendations (Table 2). All individual qRT-PCR assays are performed with any of the following machines; ABI 7500 (Applied Biosystem, USA), CFX-96 (Bio-Rad, USA) or LightCycler 480 (Roche, Switzerland), using following cycling parameters; 45 °C for 10 min, 95 °C for 10 min, and 45 cycles of 95 °C for 15 s and 55 °C for 1 min. A negative control (no template) and CDC provided specific positive control is included in every run along with the samples. The amplification data is analyzed using machine specific softwares. The cutoff value CT < 35 is considered positive. A CT value of ≤38 showing appropriate amplification on visual check is also considered a positive result.

### Data management

A site supervisor reviews all forms for completeness and protocol deviations before sending forms to the data management center at icddr,b. Electronic (scanned) versions of all forms are also regularly saved for long-term storage. The database was designed using SQL Server 2008 and data are entered using Visual Studio 2010. A set of range and consistency checks are built into the data capture system to provide immediate feedback to data entry personnel regarding errors or inconsistent data. Double data entry is used to further reduce the rate of date entry errors.

## Statistical analysis

### Sample size and power calculations

The number of children in each of the five arms of the MDARI study is determined by the main MDIG trial. For MDARI analyses, we made several assumptions including: maximum of 260 infants in each group, but with 10 % attrition/loss prior to enrolment in MDARI; incidence rate of microbiologically confirmed ARI in the placebo group of 3 cases per child-year; incidence of clinical ARI in the placebo group of 6 cases per child-year; two-sided type 1 error rate of 0.05; power of 80 %; and effect estimate reported as a hazard ratio (HR) (risk in intervention group vs. placebo group).

We anticipate that the effect of maternal vitamin D supplementation will be incremental with increasing dose, such that the greatest effect size (vs. placebo) will be observed in the highest dose vitamin D group with postpartum continuation (group E in Fig. 1). Therefore, the primary aim and power calculation are based on the pair-wise comparison of group E vs. group A (placebo). This will test the core mechanistic hypothesis that improvements in vitamin D status via maternal vitamin D supplementation reduce the risk of ARI among infants in Dhaka. For the primary analysis, we will use a *gatekeeper approach*, whereby we will proceed to comparisons of shorter and/or lower dose groups (versus placebo) only if the primary comparison of E vs. placebo is statistically significant. For example, if E vs. placebo is significant (*p* < 0.05), then we will proceed with D vs. placebo, and so forth. This approach provides strong control of the family-wise error rate (type I error) while maintaining high power for the primary comparison [40]. Our primary analysis will also estimate hazard ratios and 95 % confidence intervals for each dose relative to placebo. We thus maintain 80 % power to address the question of whether maternal vitamin D supplementation can reduce the rate of microbiologically-confirmed acute respiratory infections, while simultaneously being able to address whether lower doses are also effective, and to investigate whether a dose–response relationship exists, thereby supporting the biological plausibility and robustness of inferences. At the assumed baseline event rate of 3 events per child-year, we will be able to detect a HR of 0.74 with 80 % power, representing a 26 % reduction in the rate of microbiologically-confirmed ARI. We believe that this is consistent with a minimum clinically-relevant reduction in risk.

In secondary analyses, we will consider aggregation of multiple vitamin D arms (e.g., group D and E), which will have the benefit of increasing the sample size; however, for the a priori primary outcome analysis, we have decided to retain the separation of the groups (according to the design of the MDIG trial) to limit the possibility of increasing a type II error if a true effect of the longer duration or higher-dose intervention were diluted by the lack of effect of the other group(s). Secondary analyses will also replicate the above analyses for clinical respiratory infections (i.e., meets clinical definition with or without microbiologic confirmation) and influenza or RSV associated infections.

### Analytic plans

The primary analysis will be conducted as an intention-to-treat analysis and will include all randomized infants who enter the postnatal follow-up period (i.e., infants born to mothers who consent during pregnancy and infants whose mothers consent postnatally). The primary aim is based on the pair-wise comparison of high dose group E vs. placebo. If the evidence indicates a difference between group E vs. placebo (*p* < 0.05), we will proceed to selected between-group comparisons versus placebo. As there is expected to be some loss to follow-up (censoring), the primary approach will employ a time-to-event (survival) analysis. Specifically, we will use the Anderson-Gill extension of the Cox proportional hazards model to enable the appropriate analysis of recurrent events (i.e., repeated episodes of infection within the same child). The primary effect measure will be hazard ratios, which in this context are the same as incidence rate ratios (IRRs). However, in contrast to some regression models that directly yield IRRs (e.g., Poisson regression) the approach has the advantage of not requiring the assumption of a constant baseline incidence rate. The models will apply jackknife estimators of the standard errors to account for correlation within infants over time [41]. This approach has a similar aim as generalized estimating equations or mixed effects models to account for the non-independence of repeated events within the same child, without requiring us to discard events from multiple episodes (as in, for example, a time-to-first-event analysis). The time unit of analysis is the study week. Following an ARI meeting clinical case definition, weekly active surveillance continues, however, the infant will not be considered ‘at risk’ of a new ARI until there has been one documented case definition free study week or more than 4 study weeks have elapsed (in cases where intervening surveillance visits were missed).

Secondary analyses will include comparing aggregated “high-dose” vitamin D groups (16,800 IU/week (Group C) and 28,000 IU/week (Group D)) versus low/no vitamin D groups (4200 IU/week (Group B) and placebo (Group A)) to optimize power for detecting smaller differences. A specific pairwise comparison between the 28,000 IU/week prenatal + postpartum group (Group E) will be compared to 28,000 IU/week without postpartum supplementation (Group D) to identify the impact of continuing vitamin D supplementation in the postpartum period.

The main secondary outcomes are confirmed infection with influenza (A or B) or RSV, clinical ARI, URTI, and LRTI (i.e., clinical case definition is met but without the need for positive microbiology). The secondary analysis will also be conducted as an intention-to-treat analysis, will include all randomized infants, and will use incidence rate ratios to quantify between group differences. For the secondary outcomes, we will use recurrent event models fitted through proportional hazards models for confirmed RSV or influenza (separately by type of infection) and for clinical ARI, URTI, and LRTI (separately by clinical category). In sensitivity analyses to test the robustness of the primary findings, we will consider adjustment for baseline covariates for which distributions differ across the intervention groups.

Given the expected non-normal distribution of the data, *S. pneumococcus* density will be reported as median values with inter-quartile range. Differences in continuous variables such as density will be assessed using the Mann–Whitney and *p* < 0.05 will be considered statistically significant. When assessing difference in density of multiple groups, a Kruskall-Wallis analysis of variance will be employed.

## Discussion

The MDARI study in Dhaka, Bangladesh leverages the infrastructure of a large randomized placebo controlled, dose-ranging trial of maternal vitamin D supplementation during pregnancy and lactation in Bangladesh to test the hypothesis that maternal vitamin D supplementation can decrease infant respiratory infections during the high risk first 6 months of infancy. If shown to reduce the risk of ARI in infancy, integration of maternal prenatal/postpartum vitamin D supplementation into antenatal care programs in South Asia may be a feasible primary preventive strategy to reduce the burden of ARI-associated morbidity and mortality in young infants.

## Trial status

Data collection is ongoing.

## Abbreviations

25 (OH) D: 25-hydroxyvitamin D
Adeno: Adenovirus
ARI: Acute respiratory infection
CDC: Centers for Disease Control and Prevention
CHW: Community health worker
CT: Cycle threshold
DNA: Deoxyribonucleic acid
HMPV: Human metapneumovirus
HPIV1: Human parainfluenza-1
HPIV2: Human parainfluenza 2
HPIV3: Human parainfluenza 3
HR: Hazards ratio
HSV-1: Herpes simplex virus-1
Ikβα: Nuclear factor of kappa light chain gene enhancer in B cells, alpha
IMCI: Integrated management of childhood illness
IRR: Incidence rate ratios
IU: International units
LMP: Last menstrual period
LRTI: Lower respiratory tract infection
MCHTI: Maternal and Child Health Training Institute
MDARI: Maternal vitamin D for acute respiratory infections in infancy
MDIG: Maternal vitamin D for infant growth
NF-κB: Nuclear factor kappa light chain enhancer of activated B cells
PCR: Polymerase chain reaction
qPCR: Real time polymerase chain reaction
qRT-PCR: Real time reverse transcriptase-polymerase chain reaction
RNA: Ribonucleic acid
RSV: Respiratory syncytial virus
SpO_2_: Peripheral oxygen saturation
URTI: Upper respiratory tract infection
WHO: World Health Organization
